# ANItools web: a web tool for fast genome comparison within multiple bacterial strains

**DOI:** 10.1093/database/baw084

**Published:** 2016-06-05

**Authors:** Na Han, Yujun Qiang, Wen Zhang

**Affiliations:** ^1^State Key Laboratory for Infectious Disease Prevention and Control, National Institute for Communicable Disease Control and Prevention, Chinese Center for Disease Control and Prevention, Beijing 102206, China; ^2^Collaborative Innovation Center for Diagnosis and Treatment of Infectious Diseases, Hangzhou 310003, China

## Abstract

**Background:** Early classification of prokaryotes was based solely on phenotypic similarities, but modern prokaryote characterization has been strongly influenced by advances in genetic methods. With the fast development of the sequencing technology, the ever increasing number of genomic sequences per species offers the possibility for developing distance determinations based on whole-genome information. The average nucleotide identity (ANI), calculated from pair-wise comparisons of all sequences shared between two given strains, has been proposed as the new metrics for bacterial species definition and classification.

**Results:** In this study, we developed the web version of ANItools (http://ani.mypathogen.cn/), which helps users directly get ANI values from online sources. A database covering ANI values of any two strains in a genus was also included (2773 strains, 1487 species and 668 genera). Importantly, ANItools web can automatically run genome comparison between the input genomic sequence and data sequences (Genus and Species levels), and generate a graphical report for ANI calculation results.

**Conclusion:** ANItools web is useful for defining the relationship between bacterial strains, further contributing to the classification and identification of bacterial species using genome data.

**Database URL:** http://ani.mypathogen.cn/

## Background

Rapid and accurate classification of bacterial isolates is the most important task in medical microbiology, especially during infectious disease outbreaks with national or global spreading threat (1). However, the current classification methods all have shortcomings at the resolution level (2), not only the methods based on phenotypic similarities and chemical characteristics, but also modern genetic methods based on fragment nucleotide sequences (16S and multilocus sequence typing [MLST]) (3–5). The molecular structure of 16S rRNA is too conserved to distinguish between closely related species (>97% similarity) (6–8). Additionally, early classification of prokaryotes was based solely on phenotypic similarities and chemical characteristics, which are to some extent affected by environmental factors, such as temperature and pH, which can cause possible biases (4). Classification using 16S rRNA and MLST methods could be also biased by one or more sequencing errors.

Most recently, the average nucleotide identity (ANI), calculated from pair-wise comparisons of all sequences shared between any two strains, has been proposed as the new metrics for bacterial species definition and classification. In 2005, Pro. Konstantinidis firstly assessed 70 related species and found ANI of the shared genes between two strains to be a robust means for comparing genetic relatedness among strains; ANI values of ∼94% were shown to correspond to the traditional 70% DNA–DNA reassociation standards of the current species definition (9–11). In 2012, using 38 strains in the genus *Acinetobacter* as a test case, Chan further proved that ANI results are congruent with the core genome phylogeny and traditional approaches, and also compatible with the existing taxonomy (12). In our previous work (2), we calculated and listed the precise ANI values of any two genome comparisons in 1226 bacterial strains, indicating that species classification based on ANI is in excellent agreement with the NCBI’s bacterial taxonomy. This work proved ANI to be useful for bacterial taxonomy, representing a powerful candidate method for the definition for existing as well as novel bacterial species (2).

Comparing with other methods, ANI analysis based on whole-genome comparison between two strains has higher resolution and can avoid the bias caused by sequence selection and errors. Even two closely related bacterial species can be distinguished based on their DNA divergence at the genomic level, and one or a few sequencing errors can be easily adjusted with the help of depth coverage of sequence reads (2). Besides ANItools, the other program is available for ANI value calculation (JSpecies) (6,13). However, the use of our previous version ANItools still requires the installation as well as that of several appended programs (such as BLAST and Hmmer) on personal computer, in addition to parameter adjustments. Additionally, no ANI database is available currently to the public for thousands of bacterial genomes. Although JSpeciesWS (13) also support a web version for calculating ANI values between several bacterial strains, the strain number limitation (a maximum of 15 genomes) hinder the possibility to get the ANI matrix on genus or species level, and there is also no phylogenetic tree result to graphically show the relationship among strains in the same genus or species. Besides ANItools, there is another ANI value calculation program available named JSpecies (6,13). However, the both tools are not as perfect as we expect. ANItools still needs to be installed locally in personal computer or sever, which certain Add-In programs like BLAST and Hmmer, are also essential for in the meanwhile, always accompanied by the related parameters set up or adjustment works. That means not so friendly to the users who has no background about IT or Bioinformatics. When it comes to JSpeciesWS (13), the first tool can be used online to calculate ANI, doesn’t require any kind of parameter set up or adjustment works. But due to the limited capacities (maximum 15) of strain number in comparison, users have no chance to get the ANI matrix on genus or species level, when they are required to analyze too many strains in the meantime. Moreover, there is no phylogenetic tree result either to graphically show the relationship among strains in the same genus or species.

Therefore, we finally programmed web version of ANItools 2.0 (http://ani.mypathogen.cn/) to get rid of all disadvantages of current tools in line with the conclusion above. ANItools web version helps users directly obtain ANI values online and increases the number of genomes examined comparing to previous Linux version. A database covering ANI values of any two strains in a genus was included in this database (2773 strains, 1487 species and 668 genera). ANItools web is useful to define the relationship of bacterial strains, and helpful for the classification and identification of bacterial species using genome data. Compared with currently available software, ANItools web reduces users’ involvement to a minimum level: only genomic sequence uploading and genus data selection are required. It can automatically run genome comparison between the input genomic and data sequences, and generate a graphical report for ANI calculation results.

## Implementation

ANItools web was built around two public programs, Glimmer 3 (14) and ANItools (2). The website interface is written in Java. ANItools web can analyze nucleotide sequence in ‘strict’ FASTA format (a first line with a sequence identifier preceded by ‘>’, followed by a second line with the sequence).

The analysis process consists of the following steps:

1. Gene prediction using Glimmer 3 (14) for the query nucleotide sequence. The parameters for the software used for CDS prediction Glimmer are -o50 -g110 -t30.
2. Acquisition of an ANI value matrix from the ANI database based on the species or genus name selected by the user.
3. Comparison of all predicted gene sequences of the query sequence with the target genome sequences using BLASTN. Target genomes are nucleotide sequences of bacterial species in a genus (if user-defined genus) or species (if defined species) from the genome database. The current genome database covers 2773 strains, 1487 species and 668 genera. The genome sequences of 2773 bacterial strains from 668 genus were downloaded from the database of National Center for Biotechnology Information (NCBI: ftp://ftp.ncbi.nlm.nih.gov/genomes/Bacteria/). All these sequences are complete genome and will be updated once every 3 months.
4. Based on BLAST results, ANI was calculated between the query sequence and each target genome. First, CDSs from the query genome were searched against the reference genome. With a BLAST match of at least 60% overall sequence identity and an alignable region >70% of their length, these alignments could be kept, and the remaining CDSs considered to be genomic specific and filtered out (9). Second, genome comparisons with total alignable region <50% of the query genome length should also be filtered out. Third, for genes with multi-alignments, only alignments with highest identical sites should be kept.
5. Acquisition of a new ANI matrix combining new ANI results and covering the query sequences and target genomes in the data. Using Trex 3 (15,16), the matrix was converted to a phylogenetic tree which represents the evolutionary relationship of the query strain with the target genomes.

## Results and Discussion

**Figure 1.**
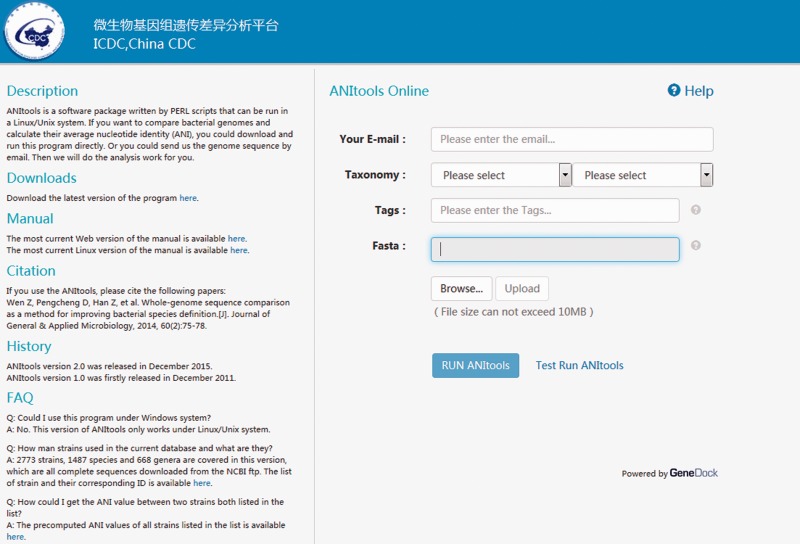
Interface of the input page.

**Figure 2.**
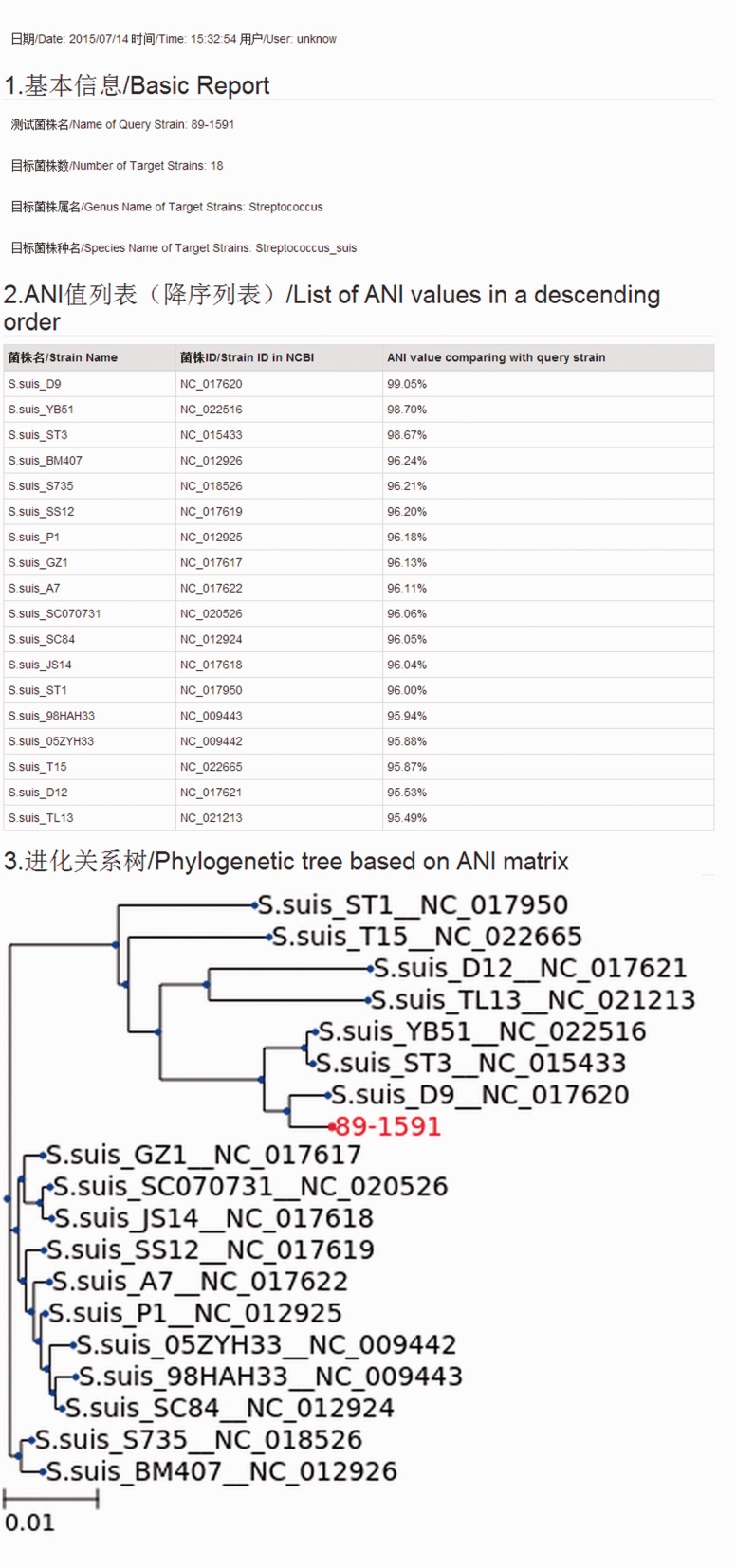
Interface of the report page. All information are shown in two languages: Chinese and English.

We have developed a web-based computational method to quickly compare bacterial strains. The use of traditional biochemistry methods and 16S sometimes only allows species distinction at the genus level. With the help of ANItools, users can obtain a list of ANI values between the query strain and every strain in the same genus, and identify the best match. Based on a large scale survey in our previous study (2), ANI values of strain pairs in the same species are usually higher than those of strain pairs from different species in a genus. Thus, the list of ANI values in the report page is useful to users in classifying previous undefined bacterial species at the genome level, combining with the next-generation sequencing (NGS) technology. Using *Streptococcus* as a model, users select ‘*Streptococcus*’ and ‘*Streptococcus suis*’ in the Taxonomy list, then upload a genome sequence in the input page (Figure 1) and click ‘Run ANItools’. Several minutes later (5–20 min), a report page is displayed (Figure 2). As shown in Figure 1, a strain sampled from a diseased pig (89–1591) in Canada with serotype 2 showed highest similarity with *S. suis* D9 (NC_017620) with serotype 7. The previous genome typing method also supports this result (1): both strains were in the Minimal Core Genome Group 4 (MCGG4) group.

A phylogenetic tree is also included in the report page, graphically representing the evolutionary relationship among bacterial strains; it is helpful for determining the pathogenic strain source in an epidemical outbreak research. Based on our previous research, even in the same pathogenic species, the pathogenicity level is also variable for the strains carrying different pathogenic genes or with variable genotype (1,17–21). Still using *Streptococcus suis* as an example, *S. suis* strains could be divided into seven groups based on minimal core genome, and Minimal Core Genome Group 1 (MCGG1) strains had higher virulence compared with those in other groups (1). Similar genetic differences within bacterial strains are also shown in our ANItools, which has the fastest calculation rate (∼10 min for result generation).

To protect the privacy of the users, the uploaded sequence and analysis results will not be kept in our database. The genome sequences in this ANI database will be updated once every 3 months for users to get more information in time.

In the current version of ANItools, the analysis is restricted to the genus or the species that users choose. And the reference genome found in elsewhere or users sequenced by themselves could not be analyzed neither. We will upgrade the ANItools as soon as possible to address these limitations in next version.

## Conclusions

To facilitate effective and fast genome comparison among bacterial strains, we have developed ANItools web, which is accessible at a website (http://ani.mypathogen.cn/). Website stability was tested by online website tools (http://www.websitepulse.com). For users interested in using ANItools on their own computer, an installation package for ANItools is also available for download.

Currently, ANItools web is being used to compare bacterial strains at the genus and species levels. This will provide further clues to define bacterial strain at the genome level and graphically represent the complex relationship among strains, which is helpful for finding a cluster of strains with high similarity (candidate pathogen strains causing an outbreak) in an epidemic study.

**Availability and** **Requirements**

**Project Name:** ANItools web.

**Project home page:** http://ani.mypathogen.cn/.

**Operating system(s):** Platform independent.

**Programming language:** Java.

**Other requirements:** Java 1.3.1 or higher.

**License:** GNU GPL.

**Any restrictions to use by non-academics:** License needed.

## Authors’ Contribution

N.H. has made contributions to acquisition of data, analysis and interpretation of data; Y.Q. have been involved in the drafting the manuscript and W.Z. contribute to the design, and write the manuscript.

## Funding

National Natural Science Foundation of China (no. 81301402), 863 Project 2014AA021505, 2013ZX10004221 and 2013ZX10004-101-002.

*Conflict of interest*. None declared.
